# Assessing Signs of Burnout Among Recently Ordained Stipendiary Anglican Curates In England

**DOI:** 10.1007/s10943-024-02205-2

**Published:** 2024-12-23

**Authors:** Greg Smith, Leslie J. Francis, Ursula McKenna, Andrew Village

**Affiliations:** 1 World Religions and Education Research Unit, Bishop Grosseteste University, Lincoln, UK; 2 Centre for Educational Development, Appraisal and Research (CEDAR), University of Warwick, Coventry, UK; 3 School of Humanities, York St John University, York, UK

**Keywords:** Balanced affect, Satisfaction in ministry, Emotional exhaustion in ministry, Anglican clergy, Curates

## Abstract

This study examines the responses of 253 recently ordained stipendiary Anglican curates in the Church of England to the Francis Burnout Inventory during their second year in ministry. The data confirm the internal consistency reliability among recently ordained clergy of the scales proposed by this measure: Scale of Emotional Exhaustion in Ministry, and Satisfaction in Ministry Scale. While positive affect is high, with 90% agreeing with the item that they gain a lot of satisfaction from fulfilling their ministry roles, negative affect is also uncomfortably high, with 35% agreeing with the item that they feel drained by fulfilling their ministry roles. It is those ordained under the age of forty who experienced the highest levels of emotional exhaustion.

## Introduction

The current pattern of Initial Ministerial Education (IME) within the Church of England has its roots in two formative reports: *Formation for ministry within a learning church* (Archbishops’ Council, 2003) and *Shaping the future* (Archbishops’ Council, 2006). This current pattern comprises two main phases. IME1, prior to ordination to the diaconate, takes place in Theological Education Institutions (TEI), sometimes in the form of full-time residency in theological colleges and sometime in the form of part-time engagement with theological courses. IME2, following ordination to the diaconate, takes places within ministry contexts, most often serving in a parish under the supervision of a training minister, supported by a diocesan structure of training provisions. Behind this pattern was the aspiration to create a seamless training experience, with a smooth transition between college and parish; a desire to measure more reliably training outcomes at the end of curacy; and a recognition that the training minister should be selected with care, since the relationship between training minister and curate is seen to be of crucial importance.

In spite of this ambition, little empirical research has focused on the experience of curates in the post-ordination period. The first major study concerning the experience of curates was reported by Burgess (1998), based on in-depth interviews conducted with 20 individuals who had trained at Lincoln Theological College between 1989 and 1994. On the basis of these interviews Burgess concluded that half of his sample had experienced an unsatisfactory relationship with their training minister, often styled training incumbent. He attributed this unsatisfactory experience to five fundamental ‘pathologies of training’: a lack of preparation by the training minister before, and feedback after a task; a lack of personal organisation and professionalism on the part of training ministers; unwillingness to share tasks or recognise curates’ abilities; the inability of training ministers to create personal relationships with a colleague; and inappropriate attitudes in an adult colleague relationship. In a subsequent study, analysing written observations from more than 50 curates, Tilley (2007) found continuing evidence for Burgess’ catalogue of pathologies, but also set alongside such evidence a wide range of positive comments made by curates regarding the relationship with their training minister.

In a subsequent study, Tilley et al. (2011) drew on psychological type theory to explore the extent to which training ministers were shaping the training experience according to their own psychological preferences or according to those of their curates. Drawing on data provided by 98 pairs of training ministers and curates, this study demonstrated that the ministry expectations placed on curates were significantly related to the psychological type profile of the training minister, but not to the psychological type profile of the curates themselves. The implication was drawn that training ministers were shaping curates in their own image, rather than developing the curates’ own preferred dispositions for ministry.

Drawing on detailed questionnaire data (including personal, religious, and psychological factors) provided by 416 pairs of curates and training ministers during 2009 and 2010, Smith and Francis (2023) addressed two core research questions. The first research question developed and tested a new measure: The Smith Attitude towards Training Incumbents Scale (SATIS; Smith & Francis, 2023). The use of the styling ‘incumbents’ reflected how this term is still widely used and recognised, while in the present paper the individual with training responsibility is styed ‘training minister’ in recognition that not all such individuals necessarily have incumbent status. The second research question explores the influence of personal, religious, and psychological characteristics of both the curate and the training minister in predicting curates’ positive attitude towards their training minister. The data demonstrated that religious factors (Catholic or Evangelical, Liberal or Conservative, Charismatic or not Charismatic) were not significant. However, both personal and psychological factors of the curates themselves were significant. The curates who rated their training minister more highly were older and more emotionally stable. Personal factors were also significant for the training ministers, but not psychological factors. The curates rated more highly the experience of working with younger training ministers. The most satisfactory experience of curacy was associated with older and emotionally stable curates working with younger training ministers.

Against this background the aim of the present study is to explore the work-related psychological wellbeing of recently ordained stipendiary Anglican curates and to assess signs of early professional burnout. First, however, it is necessary to contextualise this aim by discussing the theory and measurement of clergy burnout.

## Defining and Measuring Burnout

Recent systematic reviews of clergy health and wellbeing have confirmed the continuing theoretical and practical importance of empirical research within this area (Picornell-Gallar et al., 2024; Ruiz-Prada et al., 2021). The field of professional burnout has been significantly shaped by Christina Maslach and the Maslach Burnout Inventory (MBI; Maslach & Jackson, 1986). Both Maslach’s model of burnout and the MBI were rooted in a general understanding of the caring professions. Maslach conceptualised burnout as a three-stage progressive process, beginning with emotional exhaustion that leads to depersonalisation, that is treating clients with less care and respect. In turn depersonalisation leads to the erosion of a sense of personal accomplishment. In other words, clients who experience depersonalisation reciprocated by offering less affirmation for the services received (see Maslach, 2003). Loss of affirmation leads to self-questioning, to dissatisfaction with the self, and to dissatisfaction with the professional role.

The MBI has been introduced to clergy studies by Warner and Carter (1984) and has continued to be used in clergy studies (see Parker & Martin, 2011; Rossetti & Rhoades, 2013; Vincente-Galindo et al., 2017; Case et al., 2020; Malcolm et al., 2022; Proeschold-Bell et al., 2022). Rutledge and Francis (2004), however, were suspicious that some of the concepts and some of the language employed by the MBI might not feel right among clergy. Some initial qualitative studies interviewing clergy confirmed this suspicion and led to the development of a modified instrument, under license from the original copyright holders of the test. Further scrutiny of the original form of the Maslach Burnout Inventory led to a more extensive revision of the instrument than had been originally anticipated. The revision involved three steps. First, where necessary, the original Maslach items were redrafted to bring the concepts and language in tune with the ways in which clergy thought and spoke, recognising for example that clergy did not generally refer to those among whom they exercised ministry as ‘clients’. Second, new items were constructed to bring each of the three scales up to the same length of ten items each, whereas in the original form emotional exhaustion was measured by nine items, personal accomplishment by eight items, and depersonalisation by five items. Third, the items were re-voiced to be assessed, not on a scale of frequency, but on a scale of intensity employing the established five-point Likert scale: agree strongly, agree, not certain, disagree, and disagree strongly.

This modified form of the MBI (under license and at a cost) was employed in a series of four studies among clergy in the UK: 1,071 Anglican clergy (Rutledge & Francis, 2004), 1,276 Anglican clergy (Francis & Turton, 2004a, b), 1,468 Catholic priests (Francis, Louden, & Rutledge, 2004), and 930 Pentecostal pastors (Kay, 2000). Because all four studies had used the same measure of burnout, it was possible to examine differences among denominational groups. According to these data, Catholic priests experienced a higher level of emotional exhaustion and a higher level of depersonalisation than was the case among Anglican priests. At the same time, Catholic priests experienced a higher level of personal accomplishment than was the case among Anglican priests.

This quantity of data among different groups of clergy also enabled serious questions to be raised about Maslach’s conceptualisation and measurement of burnout. Statistically the difference between emotional exhaustion and depersonalisation was not sound. The sequential model of emotional exhaustion leading to depersonalisation and depersonalisation leading to loss of personal accomplishment, although conceptually attractive, was difficult to demonstrate empirically. Moreover, the model failed to lead to viable intervention or preventative strategies.

## Introducing the Francis Burnout Inventory

Working within the context of the Australian National Church Life Survey, Francis, Kaldor et al. (2005) introduced the Francis Burnout Inventory (FBI) that offered an alternative theoretical model of burnout. In place of the sequential model offered by Maslach, they conceptualised poor work-related psychological wellbeing and professional burnout in terms of the classic balanced affect approach to wellbeing (Bradburn, 1969). According to this approach, positive affect and negative affect operate as partially independent systems within which positive affect can ameliorate the deleterious consequences of negative affect. The preventative and therapeutic consequence of the model is that intervention strategies can be targeted to enhance positive affect more readily than the removal of the causes of negative affect.

In the FBI negative affect is assessed by the Scale of Emotional Exhaustion in Ministry (SEEM) and positive affect is assessed by the Satisfaction in Ministry Scale (SIMS). The Scale of Emotional Exhaustion in Ministry drew together items expressing lack of enthusiasm for ministry, frustration, impatience, negativity, cynicism, inflexibility, profound sadness, the sense of being drained and exhausted by the job, and withdrawal from personal engagement with the people among whom ministry is exercised. The Satisfaction in Ministry Scale drew together items expressing personal accomplishment, personal satisfaction, the sense of dealing effectively with people, really understanding and influencing people positively, being appreciated by others, deriving purpose and meaning from ministry, and being glad that they entered ministry.

The factor structure, internal consistency reliability and construct validity of the FBI has been tested in a series of studies among clergy serving in The Presbyterian Church USA (Francis, Village et al., 2011), clergy serving in the Church of England (Francis, Laycock, & Brewster, 2017; Francis, Laycock, & Ratter, 2019), Catholic priests and religious sisters serving in Italy (Francis, Laycock, & Crea, 2017; Francis, Crea, & Laycock, 2017, 2021), Anglican clergy serving in the Church in Wales (Village et al., 2018), and Methodist circuit minsters serving in Great Britain (Francis, Village, & Haley, 2023).

A growing body of studies employing the FBI among a range of different groups of clergy is currently compiling a cumulative picture of how signs of burnout vary from one group of clergy to another. An aim of the present study is to locate recently ordained stipendiary Anglican curates within the developing matrix, setting their profile alongside earlier studies.

## Age Differences in Susceptibility to Burnout

One of the best attested findings in the professional burnout literature generally, as well as within the clergy burnout literature, is that younger professionals are more susceptible to high emotional exhaustion scores than their older colleagues. For example, both Rutledge and Francis (2004) in their study of male Anglican parochial clergy and Francis, Louden and Rutledge (2004) in their study of Roman Catholic parochial clergy found higher levels of emotional exhaustion among clergy in their forties than among those aged fifty and over.

There are two theories that are generally advanced to account for this age effect. One theory, as advanced by Maslach et al. (2001), argues that clergy who suffered from emotional exhaustion at a younger age may already have left ministry, either on the grounds of ill health, or to seek alternative less stressful employment, and therefore are no longer visible within the older age group. This is a cohort effect. The other theory argues that older clergy may have learned how better to pace their work so as to avoid higher levels of emotional exhaustion later in life. This is an age effect. Randall (2007) suggested that the way to adjudicate between these two theories was to draw on data provided by clergy ordained in the same year. Randall (2007) then tested the scores recorded on the Maslach Burnout Inventory by a sample of 340 Anglican clergy in England and Wales who had all served for one year in ordained ministry. His data found the significant age effect on emotional exhaustion among this homogeneous group of clergy, suggesting that this finding is an age effect rather than a cohort effect. An aim of the present study is to replicate Randall’s analysis, this time on data generated by the Francis Burnout Inventory rather than the Maslach Burnout Inventory.

## Research Questions

Against this background, the present study is now in the position to address four specific research questions. The first research question tests the internal consistency reliability of the two measures proposed by the Francis Burnout Inventory among recently ordained stipendiary Anglican curates. The second research question positions the scores of these recently ordained stipendiary Anglican curates alongside other groups of clergy for whom data are available on the Francis Burnout Inventory to test how their displayed signs of burnout relate to the experience of longer serving clergy. The third research question examines the responses of recently ordained stipendiary Anglican curates to the 22 items of the Francis Burnout Inventory in order to map their perceived experience. The fourth research question explores the effect of age on the scores of emotional exhaustion in ministry and satisfaction in ministry among this cohort of recently ordained stipendiary Anglican curates to test whether age differences persist within this cohort.

## Method

### Procedure

Employing the method of a quantitative survey, Anglican curates who had served over a year in ordained ministry and had been listed in the *Church Times* as recently ordained as priests within Church of England or the Church in Wales were mailed a 16-page survey exploring their initial experience in ministry. Responses were completely confidential and anonymous. A 46% response rate generated 404 surveys, of which 253 were completed by candidates ordained into stipendiary ministry who had provided full data on the following measures.

## Measures

*Work-related psychological wellbeing* was assessed by the two scales proposed by the Francis Burnout Inventory (FBI; Francis, Kaldor et al., 2005). This instrument comprises two 11-item scales, the Scale of Satisfaction in Ministry (SIMS), designed to capture positive affect, and the Scale of Emotional Exhaustion in Ministry (SEEM), designed to capture negative affect. Each item is rated on a five-point Likert scale: disagree strongly (1), disagree (2), not certain (3), agree (4), and agree strongly (5). Example items concerned with satisfaction in ministry are: ‘The ministry here gives real purpose and meaning to my life’ and ‘I gain a lot of personal satisfaction from working with people in my current ministry’. Example items concerned with emotional exhaustion are: ‘I feel drained in fulfilling my ministry roles’ and ‘I am less patient with those among whom I minister than I used to be’. In their foundation paper for the FBI, drawing on a sample of 6,680 clergy from Australia, England, and New Zealand, Francis, Kaldor et al. (2005) reported the following alpha coefficients: SEEM, α = .84; SIMS, α = .84.

## Participants

The 253 participants comprised 136 men and 117 women; 121 were under the age of 40, 65 were in their forties, 65 were in their fifties, and 2 were in their sixties; 225 were White British, 16 White other, 9 Black, 2 Asian, and one preferred not to say; 69 were ordained to serve in rural ministry, 71 in urban, 91 in suburban, and 22 in urban priority areas.

## Analysis

The data were analysed by the SPSS statistical package employing the frequency and reliability, routines. Differences in SEEM and SIMS scores by age were tested using nonparametric routines due to the non-normal distribution of the SEEM and SIMS scores in this sample.

## Results

Table 1 presents the scale properties of the Scale of Emotional Exhaustion in ministry and the Satisfaction in Ministry Scale in terms of the alpha coefficient (Cronbach, 1951), the correlations between the individual items and the sum of the other ten items within the scale, and the item endorsement as the sum of the agree and agree strongly responses. These data demonstrate that both scales achieved good internal consistency reliability in terms of alpha coefficients in excess of .80, that each item contributed to a homogeneously broadly based construct, and a range of discrimination among the constituent items.

**Table 1:** Francis burnout inventory: scale properties

	*r*	%
*Scale of Emotional Exhaustion in Ministry (α = .83)*		
I feel drained by fulfilling my ministry roles	.57	35
Fatigue and irritation are part of my daily experience	.65	31
I am invaded by sadness I can’t explain	.52	12
I am feeling negative or cynical about the people with whom I work	.54	17
I always have enthusiasm for my work*	.52	73
My humour has a cynical and biting tone	.33	14
I find myself spending less and less time with those among whom I minister	.34	16
I have been discouraged by the lack of personal support for me here	.44	14
I find myself frustrated in my attempts to accomplish tasks important to me	.55	34
I am less patient with those among whom I minister than I used to be	.55	12
I am becoming less flexible in my dealings with those among whom I minister	.45	7
*Satisfaction in ministry scale (α = .86)*		
I have accomplished many worthwhile things in my current ministry	.64	89
I gain a lot of personal satisfaction from working with people in my current ministry	.66	94
I deal very effectively with the problems of the people in my current ministry	.49	61
I can easily understand how those among whom I minister feel about things	.25	75
I feel very positive about my current ministry	.68	77
I feel that my pastoral ministry has a positive influence on people’s lives	.50	95
I feel that my teaching ministry has a positive influence on people’s faith	.78	88
I feel that my ministry is really appreciated by people	.52	92
I am really glad that I entered the ministry	.65	91
The ministry here gives real purpose and meaning to my life	.61	81
I gain a lot of personal satisfaction from fulfilling my ministry roles	.71	90

*This item has been reverse coded to compute the correlations, but not the percentage endorsement. *r* = correlation between individual items and the sum of the remaining items

Tables 2 and 3 situate the mean scale scores recorded by the sample of recently ordained stipendiary Anglican curates on the Emotional Exhaustion in Ministry Scale (Table 2) and the Satisfaction in Ministry Scale (Table 3) alongside the scores recorded by nine other groups of clergy. In terms of emotional exhaustion in ministry, these recently ordained clergy are positioned fourth in the table. Their overall level of emotional exhaustion is already comparable with that of some groups of longer serving clergy. In terms of satisfaction in ministry, these recently ordained clergy are positioned first in the table. Their overall level of satisfaction in ministry is alongside the most satisfied group of longer serving clergy.

**Table 2:** Mean scores of Emotional Exhaustion in Ministry Scale

	*N*	Mean	*SD*
1. Australian clergywomen^e^	212	24.3	5.9
2. Newfrontiers lead elders^f^	134	25.3	6.9
3. Catholic priests in Italy^h^	155	25.5	6.9
4. Church of England curates^j^	253	25.8	6.8
5. Australia, England and New Zealand^b^	3715	26.0	6.5
6. Church of England clergywomen^c^	874	27.6	6.6
7. United States of America^a^	748	27.8	7.9
8. Church in Wales clergymen^g^	266	28.2	7.4
9. Methodist circuit ministers^i^	803	29.4	6.9
10. Church of England clergy^d^	521	29.6	7.4

^a^From Francis, Wulff, and Robbins (2008)

^b^From Francis, Robbins et al. (2009)

^c^From Robbins and Francis (2010)

^d^From Brewster et al. (2011)

^e^From Robbins et al. (2012)

^f^From Francis, Gubb, and Robbins (2012)

^g^From Francis, Payne, and Robbins (2013)

^h^From Francis and Crea (2015)

^i^From Francis, Haley, and McKenna (2023)

^j^The present study

**Table 3:** Mean scores of Satisfaction in Ministry Scale

	*N*	Mean	*SD*
1. Church of England curates^i^	253	45.3	5.1
2. Newfrontiers lead elders^f^	134	45.2	4.6
3. United States of America^a^	748	44.5	5.7
4. Australian clergywomen^e^	212	44.2	4.5
5. Church of England clergywomen^c^	874	43.7	4.5
6. Australia, England and New Zealand^b^	3715	43.2	4.9
7. Catholic priests in Italy^h^	155	42.6	5.1
8. Church in Wales clergymen^g^	266	42.1	5.1
9. Methodist circuit ministers^i^	803	41.6	5.3
10. Church of England clergy^d^	521	39.5	4.9

^a^From Francis, Wulff, and Robbins (2008)

^b^From Francis, Robbins et al. (2009)

^c^From Robbins and Francis (2010)

^d^From Brewster et al. (2011)

^e^From Robbins et al. (2012)

^f^From Francis, Gubb, and Robbins (2012)

^g^From Francis, Payne, and Robbins (2013)

^h^From Francis and Crea (2015)

^i^From Francis, Haley, and McKenna (2023)

^j^The present study

Table 4 addresses the fourth research question by assessing the connection between age and scores recorded in the two indices of emotional exhaustion in ministry and satisfaction in ministry. The SEEMS and SIMS scales were not quite normally distributed (SEEMS: skewness = 0.29 (SD = 0.15); kurtosis = 0.07 (SD = 0.35), Kolmogorov–Smirnov statistic = 0.06, *p* < 0.05; SIMS: skewness = −1.12 (SD = 0.15); kurtosis = 3.55 (SD = 0.31), Kolmogorov–Smirnov statistic = 0.10, *p* < .001), so the nonparametric Kruskal–Wallis test for independent samples was used to test for differences in the distributions by age. This indicated significantly higher SEEMS scores for curates under 40 compared with older age groups, but no significant difference for SIMS scores.

**Table 4:** Mean burnout scores by age

Age group	*N*	SEEM		SIMS	
		*M*	*SD*	*M*	*SD*
Under 40	121	27.7	7.0	44.4	5.8
40–49	65	23.9	5.5	46.4	3.8
50 and over	67	24.4	6.6	45.9	4.7
H		17.9^***^		4.4 NS	

NS, not significant; H, Kruskal–Wallis statistic, adjusted for ties

^***^p < .001

## Discussion and Conclusion

Drawing on data provided by 253 recently ordained stipendiary Anglican curates who completed the Francis Burnout Inventory, this study set out to address four specific research questions. The first research question tested the internal consistency reliability of the two measures proposed by the Francis Burnout Inventory, the Satisfaction in Ministry Scale and the Scale of Emotional Exhaustion in Ministry. The data reported an alpha coefficient of .83 for the Scale of Emotional Exhaustion in Ministry and an alpha coefficient of .86 for the Satisfaction in Ministry Scale. The item rest of scale correlations confirmed that each of the eleven items within the two scales contributed satisfactorily to a broad but homogeneous construct. On these grounds, the Francis Burnout Inventory can be commended as a reliable measure of work-related psychological wellbeing among recently ordained clergy, functioning in ways similar to the performance of this measure among clergy with longer service in ministry.

The second research question positioned the scores of these recently ordained stipendiary Anglican curates on the two scales of emotional exhaustion in ministry and satisfaction in ministry alongside the experience of nine groups of longer serving clergy, comprising: 748 clergy serving in The Presbyterian Church (USA); 3,715 clergy from various denominations serving in Australia, England, and New Zealand; 874 Church of England clergywomen; 521 Church of England clergy serving in rural ministry; 212 Australian clergywomen; 134 Newfrontiers lead elders; 266 Church in Wales clergymen; 155 Catholic priests in Italy; and 803 Methodist ministers in Britain. These data demonstrated that a cohort of clergy now in their second year of ministry were reporting levels of emotional exhaustion in ministry comparable with clergy who had been serving for longer in ministry. Although still embedded within their IME2, supported by their training minister and by their diocesan structure, these newly ordained clergy were not being protected from the ‘normal’ levels of emotional exhaustion recorded by their more experienced colleagues. At the same time, these data demonstrated that a cohort of clergy now in their second year of ministry were engaging levels of satisfaction with ministry comparable with the most satisfied of their more seasoned colleagues, Newfrontiers lead elders (Francis et al., 2012).

The third research question examined the responses of recently ordained stipendiary Anglican curates to the 22 items of the Francis Burnout Inventory in order to map their perceived experience. The data demonstrated that, in terms of emotional exhaustion in ministry, around one in three of the recently ordained stipendiary curates felt drained by fulfilling their ministry roles (35%), found themselves frustrated in their attempts to accomplish tasks important to them (34%), and reported fatigue and irritation as part of their daily experience (31%). One in four could not affirm that they always have enthusiasm for their work (27%). Around one in six confessed to feeling negative or cynical about the people with whom they work (17%) and that they find themselves spending less and less time with those among whom they minister (16%). More than one in ten reported that their humour has a cynical and biting tone (14%), that they have been discouraged by the lack of personal support for them in the parish (14%), that they are invaded by sadness they cannot explain (12%), and that they are becoming less patient with those among whom they minister (12%). A smaller number felt that they were becoming less flexible in their dealings with those among whom they minister (7%).

In terms of satisfaction in ministry, nine out of every ten recently ordained stipendiary Anglican curates felt that their pastoral ministry has a positive influence on people’s lives (95%), that they gain a lot of personal satisfaction from working with people in their current ministry (94%), that they feel their ministry is really appreciated by people (92%), that they are really glad they entered the ministry (91%), and that they gain a lot of personal satisfaction from fulfilling their ministry roles (90%). Eight out of every ten felt that they have accomplished many worthwhile things in their current ministry (89%), that they felt their teaching ministry had a positive influence on people’s faith (88%), and that the ministry in their parish gave real meaning and purpose to their life (81%). Seven out of every ten felt very positive about their current ministry (77%) and that they can easily understand how those among whom they minister feel about things (75%). Six out of every ten felt that they deal very effectively with the problems of the people in their current ministry (61%).

Overall, this account of the responses to the individual items gives substance to the scale scores that suggested a high level of positive affect alongside a moderate level of negative affect. The theory of balanced affect suggests that positive affect can offset the deleterious consequences of negative affect. On that theoretical basis, these recently ordained stipendiary Anglican curates may overall be functioning quite well. The danger comes when the initial experience of positive affect begins to wear thin and the negative affect does not go away.

The fourth research question explores the effect of age on the scores of emotional exhaustion in ministry and satisfaction in ministry among a cohort of curates who were now in their second year of ministry. The data demonstrated that the age effect was strong for emotional exhaustion in ministry, but not for satisfaction in ministry. These findings give weight to the view that younger clergy experience higher levels of emotional exhaustion in ministry compared with their older colleagues ordained at the same time. This suggests that the better trajectory for older clergy may be attributed to life experience and personal maturity. The implication for those with pastoral oversight of clergy is that younger clergy may be in need of greater care and support in respect of this aspect of ministry.

A limitation with the present study is that these data were collected before the disruptive effect of the COVID-19 pandemic. Several studies have drawn attention to the impact of the pandemic on both negative affect and positive affect among clergy using the Index of Balanced Affect Change (TIBACh; Francis & Village, 2021). Consequently, there would be value in repeating the current study in the present post-COVID environment.

## Data Availability

Data are available from the corresponding author upon reasonable request.
